# Association between cervical disorders and adverse obstetric outcomes: A retrospective cohort study

**DOI:** 10.3389/fmed.2022.981405

**Published:** 2022-10-28

**Authors:** Hanxiang Sun, Xiujuan Su, Yang Liu, Shijia Huang, Xiaosong Liu, Guohua Li, Qiaoling Du

**Affiliations:** ^1^Department of Obstetrics, Shanghai First Maternity and Infant Hospital, School of Medicine, Tongji University, Shanghai, China; ^2^Clinical Research Center, Shanghai Key Laboratory of Maternal Fetal Medicine, Shanghai First Maternity and Infant Hospital, School of Medicine, Tongji University, Shanghai, China; ^3^Department of Reproductive Immunology, Shanghai First Maternity and Infant Hospital, School of Medicine, Tongji University, Shanghai, China

**Keywords:** cervical disorders, low-grade abnormal cervical cytology, high-grade abnormal cervical cytology, singleton pregnancy, premature delivery, premature rupture of membranes, low birth weight, very low birth weight

## Abstract

**Objective:**

The purpose of this study was to explore the association of cervical disorders on obstetric outcomes of singleton pregnancies in China.

**Methods:**

This hospital-based retrospective cohort study of women with live singleton births included 71,097 Chinese women. We compared the risk of adverse obstetric outcomes in different types of pregnancies with cervical disorders with those with normal cervix. Logistic regression model was used to estimate the association between cervical disorders and adverse obstetric outcomes.

**Results:**

Women with cervical disorders had a higher risk of premature delivery (10.98 vs. 4.41%), preterm premature rupture of membranes (PPROM) (3.48 vs. 1.62%), low birth weight (LBW) (7.62 vs. 2.92%) and very low birth weight (VLBW) (2.01 vs. 0.28%) than women with normal cervix. After adjusting for confounding factors, compared with women with normal cervix, women with high-grade abnormal cervical cytology are at greater risk of premature birth (adjusted OR 1.971, 95% CI: 1.302–2.983), premature rupture of membranes (PROM) (adjusted OR 1.379, 95% CI: 1.047–1.815), LBW (adjusted OR 1.790, 95% CI: 1.059–3.025), and VLBW (adjusted OR 4.519, 95% CI: 1.662–12.292) than women with low-grade abnormal cervical cytology, and women with abnormal cervical cytology after treatment had a higher risk of premature birth (adjusted OR 2.060, 95% CI: 1.348–3.147), PROM (adjusted OR 1.381, 95% CI: 1.038–1.839), PPROM (adjusted OR 1.995, 95% CI: 1.022–3.892), LBW (adjusted OR 1.801, 95% CI: 1.046–3.102), and VLBW (adjusted OR 4.868, 95% CI: 1.788–13.255) than untreated women.

**Conclusions:**

Our research showed that pregnant women with cervical disorders were more likely to have premature delivery, PPROM, LBW, and VLBW. Moreover, pregnant women with high-grade abnormal cervical cytology and abnormal cervical cytology after treatment had a higher risk of premature birth, PROM, LBW, and VLBW.

## Introduction

The function of female cervix is to act as a barrier between uterus and vagina, and keep it closed until the fetus is full-term, so that the fetus can develop and mature (1). There may be many cases of cervical disorders in pregnant women, such as abnormal cervical cytology, cervical incompetence, cervical neoplasia and history of cervical surgery, etc. It has been shown that women with cervical cytology abnormalities are more likely to have premature birth, early preterm birth, PROM, and LBW (2). Cervical incompetence is known to be an important cause of premature birth (3), and shortening of the cervix is thought to be significantly associated with premature birth (4). There are few studies on the association of cervical neoplasia and surgical history with obstetric outcomes. A Japanese study found that women who underwent cervical polypectomy during pregnancy had a higher risk of miscarriage or spontaneous premature birth than the general population (5). Premature birth is an important cause of neonatal death (6). Studies have shown that the younger the gestational age, the worse the prognosis. Respiratory distress was the most common complication, followed by patent ductus arteriosus, bronchopulmonary dysplasia, retinopathy, ventricular hemorrhage, necrotizing colitis, sepsis, etc. (7, 8).

The association between cervical cytological abnormality and adverse obstetric outcomes has been studied. Studies have shown that for women with cervical cytological abnormality, the risk of premature delivery was higher in high-grade abnormal cervical cytology than in low-grade abnormal cervical cytology (2). F Bruinsma believed that women with cervical intraepithelial neoplasia (CIN) had adverse perinatal outcomes whether they were treated or not (9). However, some studies believed that women with CIN were at greater risk of premature delivery, PPROM, LBW and other adverse outcomes after treatment (10, 11). Moreover, the studies also believed that the risk of adverse outcomes was related to the operation method, the depth of cervical resection, the volume and size of cervical tissue resection (9–12). A British meta-analysis suggested that for future pregnancies, cervical resection was more likely to have adverse outcomes than cervical ablation (13). Except for cervical cytological abnormalities, there were few studies on the association between other cervical-related abnormalities and adverse obstetric outcomes, and there was no related study in China. Therefore, we studied the association between cervical disorders and adverse obstetric outcomes in women with singleton pregnancies who have given birth at one of the advanced health facilities on maternal and fetal medicine in our country.

## Materials and methods

This was a retrospective cohort study, including women with singleton pregnancies who delivered in Shanghai First Maternity and Infant Hospital from September, 2015 to August, 2020. We reviewed basic information, including age, body mass index (BMI), whether pregnancy was obtained through assisted reproductive technology, parity, mode of delivery, birth year of newborn, etc. The data came from the electronic medical records of Shanghai First Maternity and Infant Hospital. This study was approved by the Ethics Committee of the Shanghai First Maternal and Infant Hospital, affiliated with affiliated with Tongji University. The approval reference number is KS1998. Written informed consent for participation was not required for this study in accordance with the national legislation and the institutional regulations.

### Population

**Figure 1:**
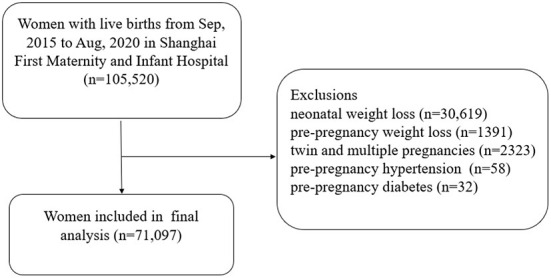
Flow chart concerning the study population.

All singleton pregnancies with live birth from September 2015 to August 2020 were retrospectively selected from the information system of Shanghai First Maternity and Infant Hospital. Records were deleted from the dataset for the following reasons: neonatal weight loss (*n* = 30,619), pre-pregnancy weight loss (*n* = 1,391), twin and multiple pregnancies (*n* = 2,323), pre-pregnancy hypertension (*n* = 58), pre-pregnancy diabetes (*n* = 32). Finally, a total of 71,097 women were included in the study (Figure 1).

### Exposure

The primary explanatory variable was cervical disorder. Cervical disorders in this study included a history of abnormalities and current abnormalities. According to whether the cervix was normal or not, we divided it into normal cervical group and abnormal cervical group. The cervical disorders in this study included cervical cytological abnormalities, cervical incompetence, cervical neoplasia (including cervical polyps, cervical fibroids and other benign lesions), and history of cervical surgery (excluding the treatment of cervical cytological abnormalities, only including history of surgery such as cervical polypectomy, etc.). We further classified cervical cytological abnormalities into low-grade abnormal cervical cytology and high-grade abnormal cervical cytology according to the degree of lesions. Low-grade abnormal cervical cytology here included atypical squamous cells of undetermined significance (ASCU-S) and CIN1, while high-grade abnormal cervical cytology included atypical squamous cells cannot exclude high-grade squamous intraepithelial lesion (ASC-H), CIN2, CIN3, squamous cell cancer and adenocarcinoma. According to treatment or not, cervical cytological abnormalities were divided into untreated group and treated group. The treated group of cervical cytology abnormality included any kind of surgical treatment, such as various resection and ablation.

### Outcomes

The outcomes of interest were adverse obstetric outcomes, including premature delivery, early premature delivery, late premature delivery, PROM, PPROM, term PROM, LBW, and VLBW. Premature delivery refers to delivery before 37 weeks of pregnancy. Early premature delivery refers to delivery between 28–34 weeks of pregnancy. Late premature delivery refers to delivery between 34–37 weeks of pregnancy. PROM refers to the rupture of membranes before the onset of labor. PPROM refers to PROM that occurred before 37 weeks. Term premature rupture of membranes refers to PROM that occurs after 37 weeks. LBW refers to the newborn's weight < 2500 g. VLBW refers to the newborn's weight < 1500 g.

### Statistical analysis

The continuous variables data were expressed as the mean ± standard deviation (X ± S). The chi-squared test was used to represent the rate of data count (percent). Logistic regression analysis was used to estimate the associations between different categories of cervical cytological abnormalities and adverse obstetric outcomes. To determine the adjusted odds ratio (OR) and 95% confidence interval (CI), adjustments were made for maternal age, pre-pregnancy BMI, parity and mode of delivery. Data analysis was performed using IBM SPSS Statistics for Windows, Version 26.0 (Armonk, NY, USA: IBM Corp). *P* < 0.05 was considered statistically significant.

## Results

A total of 71,097 pregnant women were involved in the study, of which 69,403 had normal cervix and 1,694 had cervical disorders. We divided the maternal age into four age groups. Generally speaking, the proportions of women with cervical disorders aged ≤ 24 years, 25–29 years, 30–34 years, and ≥35 years were 42 (2.48%), 522 (30.81%), 765 (45.16%), and 365 (21.55%), respectively, and compared with women with normal cervix in four age groups, the difference was statistically significant (*P* < 0.05).

There were 1,035 (61.10%) in cervical disorders group and 44,067 (63.49%) in normal cervical group with pre-pregnancy BMI of 18.5–23 kg/m^2^, and the difference was statistically significant. When BMI was ≥27.5 kg/m^2^, there were 72 (4.25%) and 2,044 (2.95%) in the abnormal and normal cervical group, respectively, and the difference between the two groups was statistically significant, too. Regarding whether the pregnancy was obtained by assisted reproductive technology, there were 176 (10.39%) in the abnormal cervical group and 4,286 (6.18%) in the normal cervical group, and there was statistical difference between the two groups. However, there was no statistical difference between the two groups in parity and delivery methods. We further divided the cervical disorders group into four categories, and found that the number of cervical cytological abnormalities was the highest (708, accounting for 41.79%), and the number of cervical surgery history was the lowest (86, accounting for 5.08%). The basic characteristics of the women included in this study are shown in Table 1.

**Table 1:** Basic characteristics of the study population by cervix.

	**Normal**	**Cervical**
	**cervix**	**disorders**
Age, y		
≤ 24	2782 (4.01)	42 (2.48) *
25–29	27467 (39.58)	522 (30.81) *
30–34	28903 (41.65)	765 (45.16) *
≥35	10251 (14.77)	365 (21.55) *
Body mass index, Kg/m^2^		
≤ 18.5	10354 (14.92)	246 (14.52)
18.5– ≤ 23	44067 (63.49)	1035 (61.10) *
23– ≤ 27.5	12938 (18.64)	341 (20.13)
>27.5	2044 (2.95)	72 (4.25) *
Assisted reproductive technology, *n* (%)		
Yes	4286 (6.18)	176 (10.39) *
No	65117 (93.82)	1518 (89.61) *
Parity, *n* (%)		
Nulliparous	51093 (73.62)	1226 (72.37)
Multiparous	18310 (26.38)	468 (27.63)
Mode of delivery, *n* (%)		
Vaginal delivery	44496 (64.11)	1105 (65.23)
Cesarean section	24907 (35.89)	589 (34.77)
Year of delivery		
2015	1457 (2.10)	29 (1.71)
2016	14897 (21.46)	320 (18.89)
2017	13978 (20.14)	413 (24.38)
2018	14052 (20.25)	344 (20.31)
2019	15803 (22.77)	345 (20.37)
2020	9127 (13.15)	243 (14.34)
Classification		
Cervical cytological abnormality	/	708 (41.79)
Cervical insufficiency	/	244 (14.40)
Cervical neoplasm	/	672 (39.67)
History of cervical surgery	/	86 (5.08)

^*^P < 0.05.

In the study of cervical disorders and adverse obstetric outcomes, we found that compared with women with normal cervix, the incidence of premature delivery (10.98 vs. 4.41%), early premature delivery (3.72 vs. 0.75%), late premature delivery (7.26 vs. 3.66%), PPROM (3.48 vs. 1.62%), LBW (7.62 vs. 2.92%), and VLBW (2.01 vs. 0.28%) in women with cervical disorders was higher, and the difference was statistically significant (*P* < 0.05). The incidence of premature delivery (including early and late premature delivery), LBW, and VLBW in women with abnormal cervical cytology, cervical incompetence and cervical neoplasia was higher than that in women with normal cervix. In addition, pregnant women with cervical incompetence were more likely to have PROM (including PPROM and term PROM). But the risk of adverse outcomes was not statistically different in women with history of cervical surgery compared with those with normal cervix (Table 2).

**Table 2:** Associations between different classifications of cervical disorders and adverse outcomes.

	**Normal cervix**	**Cervical disorders**	**Cervical disorders**			
			**Cervical cytological abnormality**	**Cervical incompetence**	**Cervical neoplasm**	**History of cervical surgery**
The total number	69403	1694	708	244	672	86
Premature birth, *n (%)*	3060 (4.41)	186 (10.98) *	54 (7.63) *	78 (31.97) *	54 (8.04) *	5 (5.81)
Early premature birth, *n (%)*	520 (0.75)	63 (3.72) *	15 (2.12) *	36 (14.75) *	13 (1.93) *	1 (1.16)
Late premature birth, *n (%)*	2540 (3.66)	123 (7.26) *	39 (5.51) *	42 (17.21) *	41 (6.10) *	4 (4.65)
Premature rupture of membranes, *n (%)*	13154 (18.95)	335 (19.78)	141 (19.92)	32 (13.11) *	144 (21.43)	22 (25.58)
Preterm premature rupture of membranes, *n (%)*	1122 (1.62)	59 (3.48) *	15 (2.12)	20 (8.20) *	23 (3.42) *	2 (2.33)
Term premature rupture of membranes, *n (%)*	12032 (17.34)	276 (16.29)	126 (17.80)	12 (4.92) *	121 (18.01)	20 (23.26)
Low birth weight, *n (%)*	2024 (2.92)	129 (7.62) *	31 (4.38) *	67 (27.46) *	32 (4.76) *	3 (3.49)
Very low birth weight, *n (%)*	196 (0.28)	34 (2.01) *	6 (0.85) *	19 (7.79) *	10 (1.49) *	0 (0)

^*^P < 0.05.

According to classifications of abnormal cervical cytology, we found that all pregnant women with abnormal cervical cytology were more likely to have premature delivery and early premature delivery than those with normal cervical cytology, regardless of the degree of cervical lesions or whether they were treated or not. In addition, pregnant women with high-grade abnormal cervical cytology and abnormal cervical cytology after treatment were more likely to have premature delivery (including early and late premature delivery), PPROM, LBW, and VLBW, and the above differences were statistically significant. Moreover, we also found that high-grade abnormal cervical cytology had a higher risk of adverse outcomes than low-grade abnormal cervical cytology and treated cervical cytological abnormalities had a higher risk of adverse outcome than untreated cervical cytological abnormalities (Table 3).

**Table 3:** Associations between different classifications of cervical cytological abnormality and adverse outcomes.

	**Normal cervix**	**Cervical cytological abnormality**		**Cervical cytological abnormality**	
		**Low–grade abnormal cervical cytology**	**High–grade abnormal cervical cytology**	**Untreated**	**Treated**
The total number	69403	417	291	440	268
Premature birth, *n (%)*	3060 (4.41)	29 (6.95) *	25 (8.59) *	30 (6.82) *	24 (8.96) *
Early premature birth, *n (%)*	520 (0.75)	7 (1.68) *	8 (2.75) *	7 (1.59) *	8 (2.99) *
Late premature birth, *n (%)*	2540 (3.66)	22 (5.28)	17 (5.84) *	23 (5.23)	16 (5.97) *
Premature rupture of membranes, *n (%)*	13154 (18.95)	73 (17.51)	68 (23.37)	78 (17.73)	63 (23.51)
Preterm premature rupture of membranes, *n (%)*	1122 (1.62)	6 (1.44)	9 (3.09) *	6 (1.36)	9 (3.36) *
Term premature rupture of membranes, *n (%)*	12032 (17.34)	67 (16.07)	59 (20.27)	72 (16.36)	54 (20.15)
Low birth weight, *n (%)*	2024 (2.92)	16 (3.84)	15 (5.15) *	17 (3.86)	14 (5.22) *
Very low birth weight, *n (%)*	196 (0.28)	2 (0.48)	4 (1.37) *	2 (0.45)	4 (1.49) *

^*^P < 0.05.

We adjusted the confounding factors for the obstetric outcomes of pregnant women with abnormal cervical cytology of different classifications, and finally found that women in both categories had a higher risk of premature delivery and early premature delivery than women with normal cervix. Compared with women with normal cervix, those with high-grade abnormal cervical cytology had a higher risk of PROM (adjusted OR 1.379, 95% CI: 1.047–1.815), LBW (adjusted OR 1.790, 95% CI: 1.059–3.025), and VLBW (adjusted OR 4.519, 95% CI: 1.662–12.292). Pregnant women with abnormal cervical cytology after treatment had a higher risk of PROM (adjusted OR 1.381, 95% CI: 1.038–1.839), PPROM (adjusted OR 1.995, 95% CI: 1.022–3.892), LBW (adjusted OR 1.801, 95% CI: 1.046–3.102), and VLBW (adjusted OR 4.868, 95% CI: 1.788–13.255) compared with those with normal cervix. In addition, for all adverse outcomes, pregnant women with high-grade abnormal cervical cytology were at greater risk than those with low-grade abnormal cervical cytology, and those with treatment were at greater risk than those with untreated cervical cytological abnormalities (Table 4).

**Table 4:** Adjusted OR (95% CI) for the associations between different classifications of cervical cytological abnormality and adverse outcomes.

	**Normal cervix**	**Cervical cytological abnormality**		**Cervical cytological abnormality**	
		**Low–grade abnormal cervical cytology**	**High–grade abnormal cervical cytology**	**Untreated**	**Treated**
The total number	69403	417	291	440	268
Premature birth, *n (%)*	Reference	1.615 (1.103–2.364)*	1.971 (1.302–2.983)*	1.580 (1.086–2.298)*	2.060 (1.348–3.147)*
Early premature birth, *n (%)*	Reference	2.294 (1.079–4.876)*	3.513 (1.725–7.152)*	2.170 (1.021–4.609)*	3.807 (1.868–7.761)*
Late premature birth, *n (%)*	Reference	1.452 (0.942–2.238)	1.586 (0.968–2.599)	1.437 (0.941–2.195)	1.621 (0.974–2.698)
Premature rupture of membranes, *n (%)*	Reference	0.899 (0.697–1.161)	1.379 (1.047–1.815)*	0.919 (0.718–1.177)	1.381 (1.038–1.839)*
Preterm premature rupture of membranes, *n (%)*	Reference	0.904 (0.403–2.029)	1.846 (0.947–3.598)	0.857 (0.382–1.923)	1.995 (1.022–3.892)*
Term premature rupture of membranes, *n (%)*	Reference	0.902 (0.693–1.175)	1.294 (0.969–1.729)	0.928 (0.719–1.198)	1.277 (0.944–1.727)
Low birth weight, *n (%)*	Reference	1.272 (0.769–2.105)	1.790 (1.059–3.025)*	1.289 (0.791–2.102)	1.801 (1.046–3.102)*
Very low birth weight, *n (%)*	Reference	1.707 (0.422–6.905)	4.519 (1.662–12.292)*	1.619 (0.400–6.550)	4.868 (1.788–13.255)*

^*^P < 0.05.

Adjusted for maternal age, pre–pregnancy BMI, parity (nulliparous, multiparous) and mode of delivery (vaginal delivery, cesarean section).

## Discussion

In a cohort study of 71,097 women in Shanghai, China, women with normal cervix and cervical disorders accounted for 97.62 and 2.38% of singleton pregnancies, respectively. Our results showed that pregnant women with cervical disorders were more likely to have premature birth (both early and late premature birth), PPROM, LBW, and VLBW. When classifying abnormal cervical cytology, after adjusting confounding factors, we found that pregnant women with abnormal cervical cytology with two different classification methods both had higher risk of premature delivery and early premature delivery than women with normal cervix. Moreover, high-grade abnormal cervical cytology women had a higher risk of adverse obstetric outcomes than low-grade abnormal cervical cytology women, and women with abnormal cervical cytology after treatment had a higher risk of adverse obstetric outcomes than untreated pregnant women.

At present, there were few studies on the association between cervical disorders and adverse obstetric outcomes in China. A Swedish study showed that women with cervical cytological abnormalities were more likely to have premature birth, early premature birth, PROM, and LBW (2), which was very similar to our findings. A cohort study in United Kingdom found that women with CIN3 had a higher risk of premature delivery and PPROM than the general population (14). Other studies have also shown that pregnant women with CIN were more likely to have premature delivery (13, 15). As we all know, cervical incompetence is an important cause of premature delivery. Joy Vink et al. reported that cervical incompetence, premature delivery and PPROM were closely related, and they were mutually causal and inseparable (3). This was consistent with our research results, because our research found that pregnant women with cervical incompetence had a higher risk of premature delivery, PROM and PPROM. This also led to a higher incidence of LBW and VLBW. According to a Japanese study, cervical polyps in early pregnancy were associated with a high risk of premature delivery and late abortion (16), which was similar to our results. We found that pregnant women with cervical neoplasia had a higher risk of premature delivery, PPROM, LBW, and VLBW. History of cervical surgery in this study referred to a history of cervix-related surgery prior to pregnancy, but excluded various excision and ablation procedures for cervical cytological abnormalities. Our results did not find that the history of cervical surgery was related to premature delivery, PROM, LBW and other adverse outcomes. Studies have shown that pregnant women had a higher risk of miscarriage and premature delivery after cervical polypectomy during pregnancy, so it was suggested that cervical polypectomy was not recommended during pregnancy unless it is suspected that the polyp is malignant (17). Tagrid Jar-Allah et al. believed that the risk of premature delivery was higher in high-grade abnormal cervical cytology than in low-grade abnormal cervical cytology (2). This was consistent with our research results. We found that compared with pregnant women with normal cervix, women with high-grade abnormal cervical cytology had more adverse outcomes and higher risk than those with low-grade abnormal cervical cytology. A meta-analysis showed that for women with CIN, there was no statistical difference in women's fertility and abortion rate in the first trimester, regardless of whether they were treated or not, but cervical treatment was related to the increased risk of abortion in the second trimester (18). Johanna Wiik et al. found that the risk of premature delivery and PROM was higher after CIN treatment (19). Other studies also believed that CIN treatment was related to the increased risk of premature delivery and PROM, and different surgical methods were also related to the risk of adverse outcomes (20, 21). It was also considered that for women with CIN, the depth of cervical resection and the volume of cervical tissue resection were all related to the increased risk of premature delivery (22, 23). A Danish study found that the risk of premature delivery increased 10 times for women who received cervical conization twice (24). However, some studies have found that the volume or depth of cervical tissue resection had nothing to do with the increase of premature delivery (25, 26). Therefore, more clinical studies are needed to reach a consistent conclusion on this issue.

The mechanism by which premature birth was more likely to occur in women with treated cervical cytological abnormalities than in untreated women is unclear. It has been shown that after partial cervical resection, the total collagen in the extracellular matrix of the cervix during regeneration decreased, and the tensile strength decreased, which led to the increased risk of premature delivery (27). Type I collagen in cervix was more in early pregnancy and less in late pregnancy, suggesting that type I collagen played an important role in maintaining pregnancy. Masaaki Iwahashi et al. believed that the amount of type I collagen in the cervix decreased after treatment, which led to the premature maturity of the cervix and ultimately leading to premature delivery (28). It was speculated that the vaginal microenvironment after partial cervical tissue removal was affected, so the defense mechanism in the face of foreign invasion was affected, ultimately leading to premature delivery (29). Compared with pregnant women with low-grade abnormal cervical cytology, women with high-grade abnormal cervical cytology were more likely to have premature birth, which may be due to the fact that most women diagnosed with high-grade abnormal cervical cytology have been treated, while women with low-grade abnormal cervical cytology may choose conservative observation. Studies have shown that the volume of cervical tissue removed affected cervical regeneration (30), so pregnant women with high-grade abnormal cervical cytology had a higher risk of premature delivery.

The advantages of our study included that the content of the study was comprehensive and the amount of data was large. Second, the conclusions of our study had a certain guiding effect on clinic, that was, for pregnant women with cervical disorders, especially those with high-grade abnormal cervical cytology and abnormal cervical cytology after treatment, obstetricians should inform them of the risk of adverse outcomes, such as premature delivery, and strengthen pregnancy supervision for them. In addition, there were some limitations in our study. First, this was a retrospective study. With incomplete information on the specific surgical modalities for the treatment of cervical cytological abnormalities and the depth and volume of cervical resection, we did not make further analysis. Second, we did not take patients with sexually transmitted infections (STI) into consideration and STI is a well-known cause of adverse obstetrical outcomes, so this was a limitation of this article. What's more, for pregnancy complications and medication during pregnancy, they may indeed have adverse effects on maternal-fetal outcomes, so this was another limitation of this study. Finally, we did not further study whether there was an association between the time after treatment of cervical cytology abnormality and the occurrence of adverse obstetric outcomes.

## Conclusion

Our study showed that women with cervical disorders were more likely to have premature delivery, PPROM, LBW, and VLBW. Moreover, pregnant women with high-grade abnormal cervical cytology and abnormal cervical cytology after treatment had a higher risk of premature birth, PROM, LBW, and VLBW.

## Data availability statement

The datasets used and analyzed during the current study are available from the corresponding author on reasonable request.

## Ethics statement

The studies involving human participants were reviewed and approved by the Ethics Committee of the Shanghai First Maternal and Infant Hospital, affiliated with Tongji University School of Medicine. The approval reference number is KS1998. The ethics committee waived the requirement of written informed consent for participation.

## Author contributions

HS drafted the manuscript. HS and QD analyzed and interpreted the data. XS, YL, and GL researched data. SH and XL conducted statistical analysis and critically revised the manuscript of important content. All authors were involved in writing of the paper and had final approval of the submitted and published versions.

## Funding

This work was supported by Shanghai Science and Technology Commission (Grant No. 20Y11907900) and Pudong Municipal Health Commission (Grant No. PW2019D-9).

## Conflict of interest

The authors declare that the research was conducted in the absence of any commercial or financial relationships that could be construed as a potential conflict of interest.

## Publisher's note

All claims expressed in this article are solely those of the authors and do not necessarily represent those of their affiliated organizations, or those of the publisher, the editors and the reviewers. Any product that may be evaluated in this article, or claim that may be made by its manufacturer, is not guaranteed or endorsed by the publisher.
